# Racial disparities in pediatric spinal fusion surgery affect perioperative outcomes: a national multicenter study

**DOI:** 10.1007/s43390-025-01094-z

**Published:** 2025-06-06

**Authors:** Alden H. Newcomb, Haseeb E. Goheer, Christopher G. Hendrix, Amanda W. Hayes, W. Garret Burks, Jonathan J. Carmouche

**Affiliations:** 1 Department of Orthopaedic Surgery, Institute for Orthopaedics and Neurosciences, Carilion Clinic, 2331 Franklin Road Southwest, Roanoke, VA 24014 USA; 2 Virginia Tech Carilion School of Medicine, 2 Riverside Circle, Roanoke, VA 24016 USA; 3 Department of Orthopaedic Surgery, University of Texas at Austin, 1601 Trinity Street, Austin, TX 78712 USA; 4 Department of Orthopedics & Sports Medicine, Houston Methodist Hospital, 18123 Upper Bay Road, Houston, Texas 77058 USA

**Keywords:** Racial disparities, Pediatric, Spinal deformity, Complication, Scoliosis

## Abstract

**Purpose:**

The aim of this study was to evaluate the effect of race and ethnic differences in perioperative outcomes and short-term complications in patients undergoing pediatric spinal fusion surgery.

**Methods:**

A retrospective cohort study was performed using prospectively collected data from the American College of Surgeons National Surgical Quality Improvement Program (ACS–NSQIP-Pediatric) Pediatric database merged with the Pediatric Spinal Fusion Procedure Targeted database from 2016 to 2022 to identify pediatric patients under 18 years who had undergone any spinal fusion procedure for scoliosis using Common Procedural Terminology codes. The study population was divided into four cohorts (1) White (2) Black (3) Asian and (4) Other or Unknown. One-way ANOVA for continuous variables and chi-square tests for categorical variables were used to identify differences in perioperative variables between the four groups. Multivariable logistic regression analysis assessed the effect of race on perioperative surgical and medical complications, extended hospital length of stay, and intensive care unit stay (ICU). Significance was defined as *p* < 0.05.

**Results:**

A total of 39,666 pediatric spinal fusion patients were identified between 2016 and 2022, of which 25, 521 were White, 6007 were Black, 1342 were Asian, and 6796 were unknown or other. Black and Asian patients experienced significantly higher rates of postoperative medical complications at 75.70 and 74.52%, compared with 69.03% for White patients (*p* < 0.001). Both Black [OR: 1.383, 95% CI (1.292–1.481)] and Asian [OR: 1.320, 95% CI (1.157–1.509)] patients had an independently increased risk for medical complications, whereas only Black patients had an increased risk for ICU stay [OR: 1.222, 95% CI (1.143–1.306)] complications following a multivariate logistic regression analysis (*p* < 0.001).

**Conclusions:**

This study provides evidence of racial disparities in outcomes after pediatric spine surgery, even after controlling for demographic and health factors. Pediatric Black and Asian patients undergoing pediatric spinal fusion have a significantly higher risk of postoperative medical complications compared with White patients. These findings emphasize the need to focus on identifying the root cause and ways to reduce racial disparities in pediatric spine surgery. The present study brings awareness to the disparity in the pediatric spine population and is useful as we work towards the reduction in such disparities and their root causes.

**Level of evidence:**

Level IV.

## Introduction

Racial disparities in healthcare are ubiquitous and transcend specialties. Non-white patients, particularly Black Americans, have been shown to have worse outcomes, higher complication rates, and longer postoperative length of stay following spine surgery. The root causation of these disparities is multifactorial and has not been fully elucidated. However, health insurance coverage, appropriate specialty access, and provider implicit biases have been shown to contribute [1].

Prior studies have shown that these disparities impact the pediatric population significantly, and that non-white pediatric patients seeking orthopedic care are especially vulnerable. For example, black pediatric patients with a closed forearm fracture were 43% less likely to be treated surgically and 35% less likely to be treated surgically following open forearm fractures than White patients [2]. Data on racial disparities in adult spine surgery has been well-documented, but there exists a paucity of data with respect to pediatric spine surgery [1, 3–5]. In a single-center retrospective review of 421 cases of adolescent idiopathic scoliosis, Thornley et al. showed that Black patients had higher rates of missed pre- and postoperative appointments and 41% higher rates of 90-day returns to the emergency department [5]. To date, there is no published data evaluating the association of race with post-surgical outcomes in the pediatric population after scoliosis surgery on a national database level.

The aim of this study was to determine if an association exists between race and 30-day outcomes following surgical treatment of scoliosis in pediatric patients. We hypothesized that differences exist between White and Non-White groups with respect to preoperative comorbidities, postoperative complications, and length of stay with higher rates observed in the Non-White cohort.

## Materials and methods

### Study design and data source

This is a retrospective study of patients from the American College of Surgeons National Surgical Quality Improvement Program Pediatric (ACS–NSQIP-Pediatric) and ACS–NSQIP-Pediatric Pediatric Spinal Fusion Procedure Targeted database. The ACS–NSQIP-Pediatric, is a prospective, risk-adjusted, and multi-institutional program that provides comprehensive procedure-specific outcomes from 150 institutions across the United States. The program includes over one hundred variables related to patient demographics, patient risk factors, preoperative laboratory values, procedural details, and 30-day complications. The database has regular audits along with participating institutions having certified clinical reviewers responsible for verifying the authenticity of the data [6]. The database is deidentified, and the study was exempt from institutional review board approval.

### Study inclusion and exclusion

**Fig. 1:**
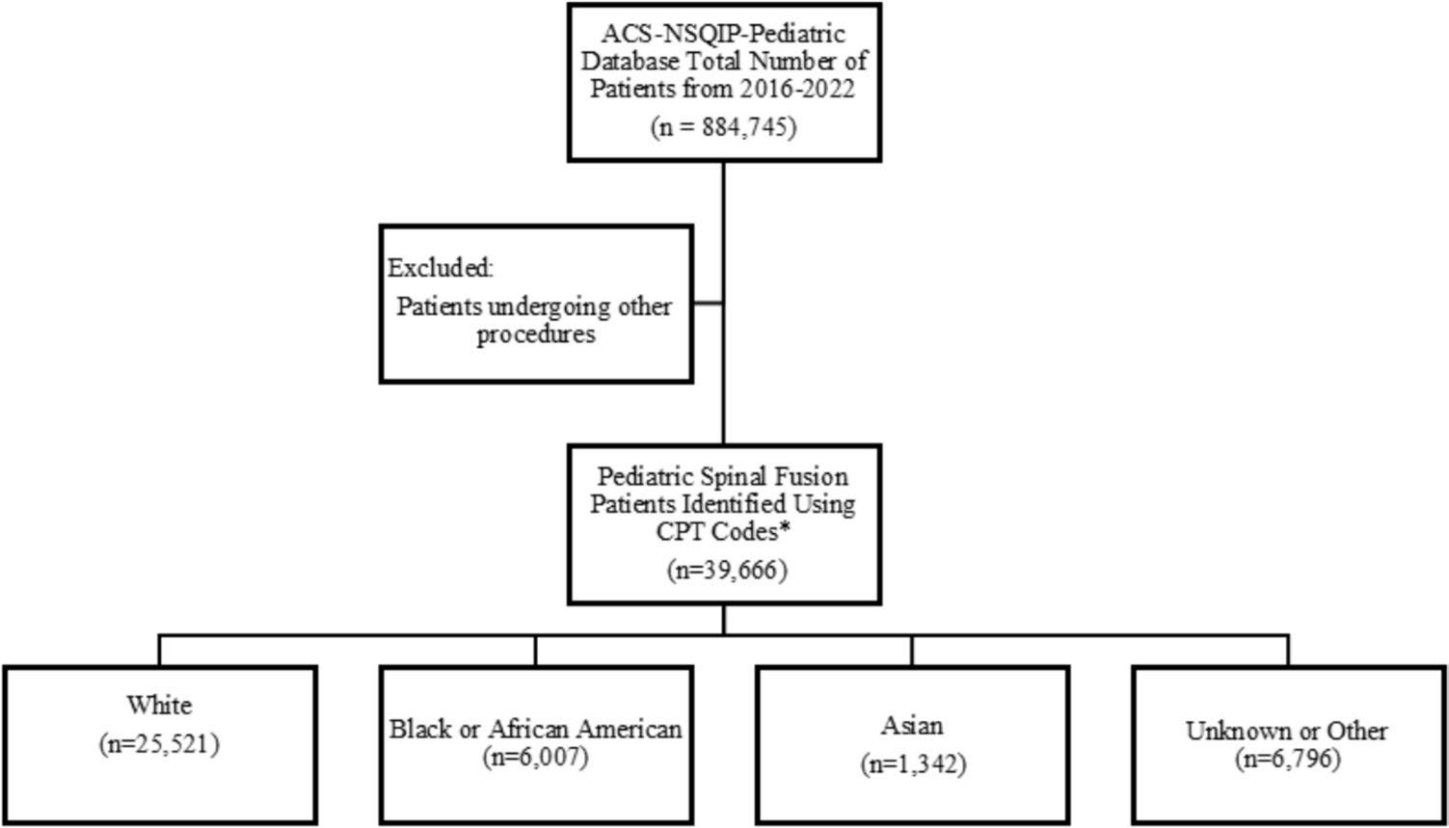
Flow diagram of the cohort selection process. *CPT codes for pediatric spinal fusion included 22800, 22802, 22804, 22808, 22810, and 22812

Data from 2016 to 2022 were reviewed to collect data on patients 18 years of age or younger undergoing pediatric spinal fusion procedures (Fig. 1). Patients were identified using the Current Procedural Terminology codes 22800 (Arthrodesis, posterior, for spinal deformity, with or without cast; up to 6 vertebral segments), 22802 (Arthrodesis, for spinal deformity; 7 to 12 vertebral segments), 22804 (Arthrodesis, for spinal deformity; 13 or more vertebral segments), 22808 (Arthrodesis, anterior, for spinal deformity; 4 to 7 vertebral segments), 22810 (Arthrodesis, anterior, for spinal deformity, with or without case; 4 to 7 vertebral segments), and 22812 (Osteotomy of spine, posterior or posterolateral approach, one vertebral segment; thoracic). Using unique case IDs, the ACS–NSQIP-Pediatric data set was merged with the ACS–NSQIP-Pediatric Spinal Fusion Procedure Targeted data set. Patients not found in both data sets, missing demographic or clinical data, or undergoing emergent procedures were excluded from the study.

### Study cohorts

Patient variables and outcomes were stratified based on race reported in the database: (1) White; (2) Black or African American; (3) Asian (A); and (4) other or unknown. Patients classified as American Indian or Alaska Native, Native Hawaiian or Other Pacific Islander, race combinations with low frequency, and patients of unknown background in the ACS–NSQIP-Pediatric data set were grouped into the other or unknown group. The race variable in the ACS–NSQIP-Pediatric was based on patients’ self-identification into one of the predefined racial identities.

### Patient variables and outcomes

Clinically relevant variables to this study from the ACS–NSQIP-Pediatric database were selected. These variables were demographic factors, intraoperative data, and postoperative outcomes within the first 30 postoperative days. Operative outcomes considered relevant to this study included surgical complications (superficial incisional surgical site infection (SSI), deep incisional SSI, organ space SSI, and wound disruption), medical complications (pneumonia, unplanned Intubation, renal insufficiency, acute renal failure, urinary tract infection (UTI), coma > 24 h, stroke or intracranial hemorrhage, seizure, nerve injury, cardiac arrest requiring CPR, bleeding/transfusions, vein thrombosis, Clostridioides difficile (c.diff) colitis, sepsis, and reoperation), extended hospital length of stay, and intensive care unit (ICU) stay.

### Statistical analysis

Differences in pre- and peri-operative variables across patient racial groups were assessed via one-way analysis of variance (ANOVA) for continuous variables and chi-square tests for categorical variables. Multivariate logistic regression analysis was used to determine independent associations for medical and surgical complications within 30 days. Significant characteristics identified in univariate analysis were controlled for in multivariate logistic regression analyses to assess the independent associations between race group and surgical complications, medical complications, intensive care unit stay, and extended hospital length of stay. The characteristics controlled for included: age, sex, surgical approach, scoliosis type, ventilator dependence, asthma, chronic lung disease, tracheostomy, esophageal or intestinal disease, history of cardiac surgery, cardiac risk factors, developmental delay, pulmonary structural abnormalities, seizure, cerebral palsy, neuromuscular disorder, intraventricular hemorrhage, nutritional support, sepsis prior to surgery, childhood malignancy, American Society of Anesthesiology (ASA) class, laparoscopic/minimally invasive surgery, type of fusion, and operative time.

The quantity and proportion of pediatric spinal fusion procedures and postoperative complications were listed by race group. Significance was defined as *p* < 0.05. All statistical analyses were performed using RStudio (R, version 4.2.2, Boston, MA, USA). American College of Surgeons National Surgical Quality Improvement Program and the hospitals participating in the ACS NSQIP are the source of the data used herein; they have not verified and are not responsible for the statistical validity of the data analysis or the conclusions derived by the authors.

## Results

### The overall pediatric spinal fusion population and race

A total of 39,666 pediatric spinal fusion patients were identified between 2016 and 2022, of which 25, 521 were White, 6007 were Black, 1342 were Asian, and 6796 were unknown or other. Black patients were more often younger (13.13 years vs. 13.38 years; *p* < 0.001), male (33.92% vs. 32.30%; *p* < 0.001), idiopathic scoliosis type (70.50% vs. 63.09%; *p* < 0.001), with a history of asthma (10.57% vs. 7.52%; *p* < 0.001), and history of cerebral palsy (13.18% vs. 9.03%; *p* < 0.001) compared to White patients (Table 1). Asian patients were more often female (71.54% vs. 67.70%; *p* < 0.001) with hematologic disorders (3.06% vs. 2.86%; *p* = 0.047) and neuromuscular disorders (22.43% vs. 21.51%; *p* = 0.017) compared to White patients. Significant differences in comorbidities between the four groups were observed (Table 1).

**Table 1:** Demographics variables and comorbidities in patients undergoing pediatric spinal fusion (*N* = 39,666)

	White *n* = 25,521	Black or African American *n* = 6007	Asian *n* = 1342	Unknown or other *N* = 6796	*p*
Age	13.38 (2.644)	13.13 (2.62)	13.21 (2.66)	13.28 (2.78)	< 0.001
Sex					
Female	67.70%	66.08%	71.54%	69.68%	< 0.001
Male	32.30%	33.92%	28.46%	30.32%	
Surgical approach					
Anterior	2.33%	1.43%	4.10%	4.24%	< 0.001
Posterior	97.67%	98.57%	95.90%	95.76%	
Scoliosis					
Idiopathic	63.09%	70.50%	62.30%	61.21%	< 0.001
Congenital/structural	6.46%	5.69%	9.24%	7.56%	
Kyphosis	5.53%	1.78%	2.83%	4.81%	
Neuromuscular	19.87%	18.48%	19.21%	19.88%	
Syndromic	3.95%	2.61%	4.84%	4.89%	
Unable to classify	1.11%	0.93%	1.56%	2.65%	
Ventilator dependence	2.48%	2.00%	1.64%	1.97%	0.007
Asthma	7.52%	10.57%	7.23%	7.24%	< 0.001
Chronic lung disease	4.94%	5.21%	4.99%	6.27%	< 0.001
Oxygen support	1.90%	1.63%	1.27%	1.72%	0.188
Tracheostomy	1.87%	2.38%	1.27%	1.63%	0.004
Esophageal, gastric, or intestinal disease	10.37%	8.92%	5.89%	8.18%	< 0.001
History of cardiac surgery	4.31%	3.36%	4.69%	4.00%	0.006
Cardiac risk factors					
Severe cardiac risk factors	0.56%	0.32%	0.75%	0.37%	< 0.001
Major cardiac risk factors	4.33%	3.31%	4.92%	4.16%	
Minor cardiac risk factors	5.24%	4.34%	5.29%	4.87%	
No cardiac risk factors	89.88%	92.03%	89.05%	90.60%	
Developmental delay	23.62%	23.24%	19.97%	23.48%	0.022
Pulmonary structural abnormalities	7.35%	5.93%	7.6%	6.81%	< 0.001
Seizure	10.36%	12.22%	7.45%	10.65%	< 0.001
Cerebral Palsy	9.03%	13.18%	8.57%	9.84%	< 0.001
Structural CNS abnormalities	13.91%	13.27%	13.19%	13.43%	0.463
Neuromuscular disorder	21.51%	19.78%	22.43%	21.51%	0.017
Intraventricular hemorrhage	0.92%	1.17%	0.60%	0.66%	0.014
Steroid use	1.00%	0.88%	0.67%	0.93%	0.578
Ostomy	10.92%	11.00%	9.54%	10.87%	0.451
Nutritional support	8.61%	8.97%	6.71%	9.03%	0.037
Sepsis prior to surgery	0.48%	0.83%	0.15%	1.13%	< 0.001
Hematologic disorders	2.86%	3.55%	3.06%	2.91%	0.047
Inotropic support at time of surgery	0.42%	0.37%	0.6%	0.49%	0.583
CPR within 7 days of surgery	0.04%	0.02%	0.00%	0.01%	0.489
Transfusions	0.16%	0.30%	0.15%	0.16%	0.159
Childhood malignancy	1.13%	0.6%	0.82%	1.13%	0.002
ASA class					
I: no disturbance	15.21%	16.45%	23.77%	17.95%	< 0.001
II: mild disturbance	51.52%	53.89%	45.83%	43.98%	
III: severe disturbance	30.88%	27.33%	27.94%	33.87%	
IV: life threatening	2.36%	2.31%	2.31%	3.33%	
V: moribund	0.00%	0.00%	0.15%	0.87%	
Work RVU (mean)	32.63 (5.32)	32.84 (4.97)	32.34 (5.42)	32.71 (5.41)	0.336
Laparoscopic/minimally invasive surgery	2.12%	1.80%	0.82%	1.57%	< 0.001
Type of fusion					
Primary	89.94%	92.36%	90.69%	89.91%	< 0.001
Revision	10.06%	7.64%	9.31%	10.09%	
Operative time (min)	284.4 (106.4)	290.7 (102.2)	304.8 (107.0)	301.6 (115.9)	< 0.001

ASA, American Society of Anesthesiologists

### Race and pediatric spinal fusion procedure characteristics

Overall Black and Asian patients experienced longer operative times (290.7 min, 304.8 min vs. 284.4 min; *p* < 0.001) and a lower prevalence of procedures using a laparoscopic or minimally invasive approach (1.80%, 0.82% vs. 2.12%; *p* < 0.001) (Table 1). Black patients had a higher prevalence of patients classified as idiopathic scoliosis (70.50.0% vs. 63.09%; *p* < 0.001) compared to White patients who had a greater proportion of scoliosis classified as neuromuscular (19.87% vs. 18.48%;* p* < 0.001). Patients in the Black, Asian, Unknown or Other cohorts had a higher prevalence of intraoperative use of antifibrinolytic (90.63%, 91.80%, 92.29% vs. 88.44%; p < 0.001), postoperative neurological deficit (1.13%, 1.57%, 2.00% vs. 1.03%; p < 0.001), and postoperative ICU stay (39.20%, 38.15%, 40.13% vs. 36.22%; p < 0.001) (Table 2), while White patients had a higher proportion of patients who had intraoperative use of neuromonitoring for spinal fusion (96.67% vs. 96.39%, 95.465, 95.20%; p < 0.001) (Table 2).

**Table 2:** Univariate analysis of pediatric spinal fusion characteristics by cohort

	White *n* = 25,521 (%)	Black or African American *n* = 6007 (%)	Asian *n* = 1342 (%)	Unknown or other *N* = 6796 (%)	*p*
Pre-operative MRI for spinal fusion	35.30	36.56	35.30	36.02	0.0147
Intensive care unit stay	36.22	39.20	38.15	40.13	< 0.001
Intraoperative use of neuromonitoring	96.67	96.39	95.46	95.20	< 0.001
Intraoperative use of antibiotics	86.44	87.30	78.39	67.04	< 0.001
Intraoperative use of antifibrinolytics	88.44	90.63	91.80	92.29	< 0.001
Postoperative neurological deficit	1.03	1.13	1.57	2.00	< 0.001

### Postoperative complications and multivariate analyses

Black and Asian patients had a higher rate of having the occurrence of one or more medical complications (75.70%, 74.52% vs. 69.03%; *p* < 0.001) relative to White patients. Black patients had higher rates of postoperative complications including pneumonia (0.87%), bleeding requiring transfusion (75.23%), C. diff colitis (0.27%), and extended length of hospital stay (2.04%) (Table 3).

**Table 3:** Univariate analysis of 30-day post-operative complications after pediatric spinal fusion by cohort

	White *n* = 25,521 (%)	Black or African American *n* = 6007 (%)	Asian *n* = 1342 (%)	Unknown or other *N* = 6796 (%)	*p*
**Surgical complications**	2.02	2.00	1.94	2.71	0.004
Superficial incisional surgical site infection	0.91	0.85	1.19	1.38	0.003
Deep incisional surgical site infection	0.78	0.78	0.45	0.93	0.2955
Organ space surgical site infection	0.35	0.37	0.37	0.40	0.947
Wound disruption	0.54	0.72	0.45	0.53	0.376
**Medical complications**	69.03	75.70	74.52	67.26	< 0.001
Pneumonia	0.87	1.03	0.89	1.25	0.032
Unplanned intubation	0.54	0.73	0.45	0.68	0.1978
Renal insufficiency	0.05	0.07	0.07	0.06	0.909
Acute renal failure	0.02	0.02	0.00	0.01	0.908
Urinary tract infection (UTI)	0.51	0.45	0.89	0.99	< 0.001
Coma > 24 h	0.01	0.00	0.00	0.01	0.812
Stroke or intracranial hemorrhage	0.02	0.03	0.00	0.00	0.508
Seizure	0.05	0.07	0.07	0.01	0.515
Nerve injury	0.33	0.22	0.52	0.22	0.013
Cardiac arrest requiring CPR	0.13	0.15	0.07	0.06	0.407
Bleeding requiring transfusion	68.57	75.23	73.85	66.67	< 0.001
Vein thrombosis	0.15	0.17	0.15	0.12	0.906
C. diff colitis	0.12	0.27	0.30	0.21	0.031
Sepsis	0.60	0.70	1.04	0.62	0.213
Reoperation	2.86	3.10	2.01	3.12	0.122
Extended length of hospital stay	2.00	2.04	2.77	3.41	< 0.001

Multivariate logistic regression was performed to determine the differential effect race on medical complications, surgical complications, ICU stay, extended length of stay, and ICU stay while adjusting for the greater comorbidity burden (Table 4). Black patients were found to have increased risk of medical complications [OR: 1.383, 95% CI (1.292–1.481); *p* < 0.001] and intensive care unit stay [OR: 1.222, 95% CI (1.143–1.306); *p* < 0.001], Asian patients also had an increased risk of medical complications [OR: 1.320, 95% CI (1.157–1.509); *p* < 0.001], postoperatively after pediatric spinal fusion. There were no statistically significant findings between race and the risk of surgical complications and extended length of hospital stay within 30 days after spinal fusion procedures.

**Table 4:** Multivariate adjusted 30-day pediatric spinal fusion postoperative outcomes among cohorts compared to white cohort

Overall cohort	Adjusted odds Ratio	95% CI		*p*
		Lower	Upper	
**Surgical complications**				
Black or African American	1.029	0.834	1.259	0.784
Asian	1.025	0.669	1.501	0.904
Unknown or Other	1.335	1.119	0.942	** < 0.001**
**Medical complications**				
Black or African American	1.383	1.292	1.481	** < 0.001**
Asian	1.320	1.157	1.509	** < 0.001**
Unknown or other	0.886	0.833	1.863	** < 0.001**
**Extended hospital LOS**				
Black or African American	1.139	0.922	1.399	0.219
Asian	1.383	0.958	1.937	0.070
Unknown or Other	1.578	1.333	1.140	**0.038**
**Intensive care unit stay**				
Black or African American	1.222	1.143	1.306	** < 0.001**
Asian	1.029	0.904	1.171	0.661
Unknown or other	1.069	1.004	1.140	**0.038**

Bold values indicate statistical significance (*p* < 0.05)

## Discussion

This study sought to elucidate how racial disparities impact perioperative outcomes in pediatric patients undergoing scoliosis surgery. Consideration of ethnoracial disparities in healthcare is imperative for the provision of equitable healthcare to all demographics. Social determinants of health (SDOH) previously demonstrated in literature to contribute to these inequalities include insurance coverage (lack of insurance or public insurance), low socioeconomic status, transportation, health literacy, cultural stigmata, and reduced access to specialty care [1, 3, 4, 7]. Non-White patients are less likely to have access to transportation, jobs with paid medical leave, and high-quality insurance [8–10]. In addition, lower socioeconomic status patients are less likely to receive referrals and have smaller specialist networks [11–13]. This analysis of the association between race and postoperative outcomes after scoliosis surgery found that Black patients had higher rates of postoperative medical complications when compared to White patients. Specifically, Black patients had higher rates of postoperative pneumonia, transfusion requirements, and C. difficile colitis. On multivariate analysis that adjusted for comorbidity burden, patients who identify as Black and Asian were independently associated with increased risk of postoperative medical complications. Black race was independently associated with increased need for ICU level of care. There were no significant differences between groups on multivariate analysis with respect to risk of surgical complications and postoperative total length of stay. Our hypothesis that an association between race and short-term outcomes after posterior spinal fusion for scoliosis in pediatric patients is supported by our data.

Consistent with previously published data by Malyavko et al., this study demonstrated a higher baseline burden of pulmonary comorbidities in Black African American patients after surgical treatment of DDH and femur fractures compared to other ethnoracial groups [14, 15]. This study showed similarly increased rates of postoperative pneumonia and unplanned reintubation in the Black patient cohort as seen following surgical treatment of developmental hip dysplasia [14]. Smaller single-institution level studies have also demonstrated racial disparities in pediatric populations after posterior spinal fusion for scoliosis. A retrospective review of 421 adolescent idiopathic scoliosis cases showed that Black patients were 66% less likely to obtain a pre-operative second opinion, miss pre- and postoperative appointments, and have a return to the emergency department within 90 days of surgery compared to White patients. This study also demonstrated a trend towards higher rates of major complications in Black patients but did not reach statistical significance [5]. Kaushal et al. examined the association of race on blood loss in pediatric posterior fusion for AIS and found that Black patients had statistically lower preoperative hemoglobin levels and longer operative times compared to White patients, but did not demonstrate a difference between racial groups in surgical blood loss [16]. Our data show statistically significant differences in operative time between racial groups, with mean operative times of Black, Asian, and Other/Unknown groups being 6, 20, and 17 min longer compared to White, respectively.

Although our data add to a growing amount of literature that consistently demonstrates poorer outcomes in minority pediatric patients, consideration must be given to the source and context. Data from the ACS–NSQIP-Pediatric database includes only short-term (30-day) outcome data, precluding drawing any sort of conclusions regarding the impact of race on long-term outcomes after posterior spinal fusion for scoliosis. The retrospective nature of the study design precludes the identification of cause–effect relationships. While demographic data were included, factors such as socioeconomic status, educational attainment, and insurance were not collected. In addition, the present analysis simplified race into broad categories which may not fully represent the diverse racial identities of patients due to limitations in the data available by the ACS–NSQIP-Pediatric database. The database also fails to define pertinent clinical information including the timing of the operation. Furthermore, preoperative imaging is not available in the database. Therefore, the authors are not able to determine the specific features of the curve including the type, magnitude, and flexibility. The ACS–NSQIP-Pediatric does not capture granular detail needed to assess equity in the availability of healthcare. This study also pooled all scoliosis surgeries into one cohort, so there may be differences in outcomes between idiopathic, neuromuscular, and congenital scoliosis. The ACS–NSQIP-Pediatric database does provide a nationwide sample of pediatric patient data, allowing access to large sample sizes. However, this study was also limited to patients who sought care within the institutions in the ACS–NSQIP-Pediatric database. Pediatric patients seeking care for scoliosis may not have been fully captured.

In conclusion, this study demonstrated higher rates of 30-day postoperative medical complications in pediatric Black patients undergoing posterior spinal fusion for scoliosis when compared to White patients. Specifically, the Black race was associated with higher rates of postoperative pneumonia, unplanned reintubation, need for transfusion, and longer operative times on univariate analysis. Further work is warranted to further elucidate the underlying causative factors, impact on long-term outcomes, and potential solutions.
